# Regression-based normative data for the D-KEFS Color-Word Interference Test in Norwegian adults ages 20–85

**DOI:** 10.1080/13854046.2023.2276967

**Published:** 2023-11-16

**Authors:** Jacob Espenes, Ingrid Myrvoll Lorentzen, Ingvild Vøllo Eliassen, Erik Hessen, Knut Waterloo, Santiago Timón-Reina, Tormod Fladby, Kristine B. Walhovd, Anders M. Fjell, Bjørn-Eivind Kirsebom

**Affiliations:** a Department of Psychology, Faculty of Health Sciences, The Arctic University of Norway, Tromsø, Norway; b Department of Neurology, University Hospital of North Norway, Tromsø, Norway; c Department of Neurology, Akershus University Hospital, Lørenskog, Norway; d Department of Psychology, University of Oslo, Oslo, Norway; e Departamento de Inteligencia Artificial, Universidad Nacional de Educación a Distancia, Madrid, Spain; f Institute of Clinical Medicine, Campus Ahus, University of Oslo, Oslo, Norway; g Center for Lifespan Changes in Brain and Cognition, University of Oslo, Norway; h Computational Radiology and Artificial Intelligence, Department of Radiology and Nuclear Medicine, Oslo University Hospital, Norway

**Keywords:** Neuropsychological tests, norms, Color-Word interference test, cWIT, norway, cross-cultural neuropsychology, executive function, D-kefs, stroop test

## Abstract

**Objective:** The Delis-Kaplan Executive Function System (D-KEFS) Color-Word-Interference Test (CWIT; AKA Stroop test) is a widely used measure of processing speed and executive function. While test materials and instructions have been translated to Norwegian, only American age-adjusted norms from D-KEFS are available in Norway. We here develop norms in a sample of 1011 Norwegians between 20 and 85 years. We provide indexes for stability over time and assess demographic adjustments applying the D-KEFS norms. **Method:** Participants were healthy Norwegian adults from Center for Lifespan Changes in Brain and Cognition (LCBC) (*n* = 899), the Dementia Disease Initiation (*n* = 77), and Oslo MCI (*n* = 35). Using regression-based norming, we estimated linear and non-linear effects of age, education, and sex on the CWIT 1-4 subtests. Stability over time was assessed with intraclass correlation coefficients (ICC). The normative adjustment of the D-KEFS norms was assessed with linear regression models. **Results:** Increasing age was associated with slower completion on all CWIT subtests in a non-linear fashion (accelerated lowering of performance with older age). Women performed better on CWIT-1&3. Higher education predicted faster completion time on CWIT-3&4. The original age-adjusted norms from D-KEFS did not adjust for sex or education. Furthermore, we observed significant, albeit small effects of age on all CWIT subtests. ICC analyses indicated moderate to good stability over time. **Conclusion:** We present demographically adjusted regression-based norms and stability indexes for the D-KEFS CWIT subtests. US D-KEFS norms may be inaccurate for Norwegians with high or low educational attainment, especially women.

## Introduction

The basic premise of Stroop tests is to measure an individual’s ability to suppress a well-learned automatic response (i.e. word reading) in favor of an unfamiliar and incongruent task (i.e. naming the printed ink color of incongruously named color names) (Rabin et al., 2005; Van der Elst et al., 2006). Inhibiting the automatic response is demanding, leading to slower speed and lower accuracy on the incongruent task. This discrepancy is referred to as the “Stroop interference effect.” While the exact nature of the cognitive processes responsible for the Stroop effect is still discussed, the effect is often regarded to measure the ability to inhibit cognitive interference and maintain focused attention (Scarpina & Tagini, 2017). The prefrontal cortex is highly involved when the Stroop test is performed (Duchek et al., 2013; Keifer & Tranel, 2013; Milham et al., 2002; Miller & Cohen, 2001), and clinical studies have shown that the Stroop interference effect is more pronounced in clinical populations, including patients with frontal lobe dysfunctions (Stuss et al., 2001), anorexia (Ferro et al., 2005), traumatic brain injury (Ben-David et al., 2011), substance use disorders (Streeter et al., 2008), mild cognitive impairment due to Parkinson’s disease (Bezdicek et al., 2015) and dementia by various etiologies (Bayard et al., 2011; Clark et al., 2012).

In cognitively healthy adults, previous research has indicated that a higher level of education is related to better test performance (Brugnolo et al., 2016; Ktaiche et al., 2022; Van der Elst et al., 2006). Consistently, young adults perform better compared to elderly (Brugnolo et al., 2016; Zalonis et al., 2009). Regarding sex differences, there are inconsistent findings with some studies reporting slight sex differences in favor of women (Magnusdottir et al., 2021; Van der Elst et al., 2006), while others find no significant difference (Brugnolo et al., 2016; Ktaiche et al., 2022). Some have found significant interaction-effects on Stroop paradigms. Van der Elst et al. (2006) reported that age-related decline was stronger for individuals with less education. On the other hand, Magnusdottir et al. (2021) found that individuals with more education exhibited a stronger age-related decline.

The Stroop test exists in several versions such as the Victoria version (Regard, 1981), the Golden version (Scarpina & Tagini, 2017) and the Color-Word Interference Test (CWIT) from the Delis-Kaplan Executive Function System (D-KEFS) (Delis et al., 2001). All tasks yield variations of the Stroop interference effect but differ in how the main outcomes are measured. The Victoria Version and Golden Version use the number of correct responses in a fixed amount of time as the outcome. In comparison, the CWIT uses time to completion on a fixed number of test items as the main outcome. Furthermore, the CWIT features a unique fourth condition called inhibition/switching, in which participants are asked to alternate between inhibition and reading color-words. This condition may be more challenging than the classic Stroop color-word inhibition task for some individuals (Lippa & Davis, 2010).

A recent review commissioned by the Norwegian Psychologist Association, the Norwegian Directorate of Health, and the Norwegian Institute of Public Health (Ryder, 2021) indicated that the D-KEFS test battery was amongst the most popular tests used by clinicians in Norway. Also, previous studies have indicated that as much as 91% of Norwegian neuropsychologists use a version of the Stroop test (Egeland et al., 2016). Ryder (2021) reports that despite its popularity, Norwegian norms, in addition to validity and reliability measures, are lacking for the D-KEFS battery. The D-KEFS battery was consequently identified as a priority for validation and norming (Ryder, 2021). To our knowledge, there are no norms outside the original American age-adjusted norms presented in the D-KEFS manual by Delis et al. (2001) available for clinicians and researchers in Norway. Thus, the main objective of this study was to investigate the effect of demographic variables on CWIT performance and provide normative data for the D-KEFS CWIT in a Norwegian sample of cognitively healthy adults. Secondly, we assess the normative adjustment of the original age-adjusted norms from D-KEFS in the same sample of cognitively healthy Norwegian adults. Lastly, for a sub-set of the sample with data from one follow-up testing we provide indexes for stability over time on the D-KEFS CWIT.

## Methods and materials

### Participants

#### Normative samples

To develop norms on the Color-Word Interference test (CWIT) we included healthy participants from three research projects in Norway: Studies from the center for lifespan changes in brain and cognition (LCBC) (*n* = 899), the dementia disease initiation study (DDI) (*n* = 77), and the Oslo MCI study (*n* = 35). Descriptive statistics from the normative sample is presented in Table 1. Joint exclusion criteria for all studies were severe somatic or psychiatric illnesses that might influence cognitive functioning. All participants underwent an interview screening for current or previous signs of neurological disorders, epilepsy, stroke, and psychiatric disorders. Participants reporting a subjective experience of cognitive decline such as memory complaints were excluded. The Mini Mental State Examination (MMSE) was used to assess global cognitive functioning and was not used to exclude participants in the current study (Table 1). Total scores on the MMSE were distributed as: 5 participants (0.5%) scored 24; 3 participants (0.3%) scored 25; 23 participants (2.4%) scored 26; and the remaining 940 participants with available MMSE (∼96.8%) scored between 27 and 30. Inclusion criterion were ages 20–85. All participants reported Norwegian as their native language and almost all participants were of European ethnicity.

**Table 1.** Descriptive statistics for the normative sample (*n* = 1011).

Variable	*Mean* (*SD*)	[Min, Max]	*Median*
Age	46.2 (19.4)	[20, 85]	43
Female *n* (%)	675 (66.8%)		
Years of education	15.5 (2.9)	[7, 23]	16
MMSE^1^	29.1 (1.1)	[24, 30]	29
CWIT-1 raw score	30.1 (5.7)	[15, 60]	29
CWIT-2 raw score	22.0 (4.3)	[13, 79]	21
CWIT-3 raw score	53.0 (14.6)	[25, 154]	50
CWIT-4 raw score	60.4 (17.3)	[28, 172]	57
Total errors CWIT-3^1^	1.0	[0, 11]	1
Total errors CWIT-4^1^	1.1	[0, 11]	1

*Note. SD* = standard deviation of the mean; *n* = count; CWIT = Color-Word Interference Test; Min = lowest score; Max = highest score; MMSE = Mini Mental State Examination; ^1^ 76 participants had missing values on errors and 40 on MMSE.

The LCBC (Fjell et al., 2019) is a multi-disciplinary research center based in Oslo, Norway aimed at investigating normal trajectories of brain and cognition across the lifespan. Healthy participants from LCBC were drawn from three longitudinal sub-projects within the LCBC; Neurocognitive development (Tamnes et al., 2013), Neurocognitive plasticity (de Lange et al., 2018), and Biological Predictors of Memory (Storsve et al., 2014). Participants were recruited through newspaper advertisements and through local Universities and workplaces. Most participants from LCBC were screened for brain abnormalities on MRI scans and participants were excluded if scans showed signs of pathology. A subset of the LCBC sample (*n* = 335) had available follow-up examinations (average test-retest interval 3.4 years) on the CWIT, allowing for test-retest analysis to assess the stability of scores over time. All healthy participants in the test-retest sample fulfilled inclusion criteria and none of the exclusion criteria at baseline testing. Participants in the test-retest sample were not excluded based on these criteria at follow-up examinations.

DDI is a Norwegian multi-center longitudinal study on early phases of Alzheimer’s Disease and other neurodegenerative diseases (Fladby et al., 2017). Inclusion criterion in the DDI study was age 40–80 years. The Oslo MCI study is the predecessor of the ongoing DDI study and followed the same study protocol as DDI. Assessments in Oslo MCI were performed between 2004 and 2012, and in DDI from 2012 to 2022. Healthy controls from DDI and Oslo MCI were either spouses of symptom group participants, volunteers recruited from advertisements in news outlets, or patients recruited at an orthopedic ward.

### Color-word interference test (CWIT) administration procedures

The CWIT consists of four subtasks. CWIT-1 requires color-naming. The participant is asked to verbally identify the color of solid-colored squares from a sheet of paper. The squares are colored red, green, or blue, and are shown in a random order for a total of 50 items. CWIT-2 requires color-reading. In this subtask, participants are shown the color names “red,” “green,” “blue” (in Norwegian “*rød*,” “*grønn*,” “*blå*”) printed in black ink. The participants are asked to read the color names one-by-one. CWIT 3 (inhibition) corresponds to the classic Stroop task, in which color names are printed with incongruent ink (e.g. “red” printed in green ink). Participants are asked to verbally identify the color of the ink, (thus inhibiting the automated response of reading the color name). CWIT-4 is the inhibition/switching condition. Again, color names are printed with incongruent ink, but approximately fifty percent of the items are enclosed within a black frame. The participant is asked to perform the same task as before (i.e. name the printed color of the ink), except for stimuli that are enclosed within the black frames. Here, the participants are instructed to read the color names. For all subtasks, the participant is asked to respond one-by-one, in succession from left to right, as quickly as possible without making errors. All subtasks are preceded by a brief untimed practice trial consisting of a 10-item sample of the pertinent subtest. The stimuli are organized on laminated sheets in A4 size. Items are arranged in 5 rows of 10 items, totaling 50 items for each subtask. Time to completion and errors are recorded. Errors are recorded as either “corrected” or “uncorrected” by the participant. Difficulty discerning colors or visual impairments impact task performance on the CWIT, and it is important for test administrators to be sensitive for any color-blindness or visual impairment in participants. Administration of the CWIT was terminated by the test administrator if participants reported difficulty discerning colors associated with color-blindness. Administration procedures and standardized instructions for all tasks are described in the D-KEFS manual (Delis, 2005; Delis et al., 2001). Standardized commercially available materials for the D-KEFS CWIT in Norwegian were purchased from Pearson Clinical Norway.

### Statistical analyses

#### Regression norming procedure

**Figure A1.**
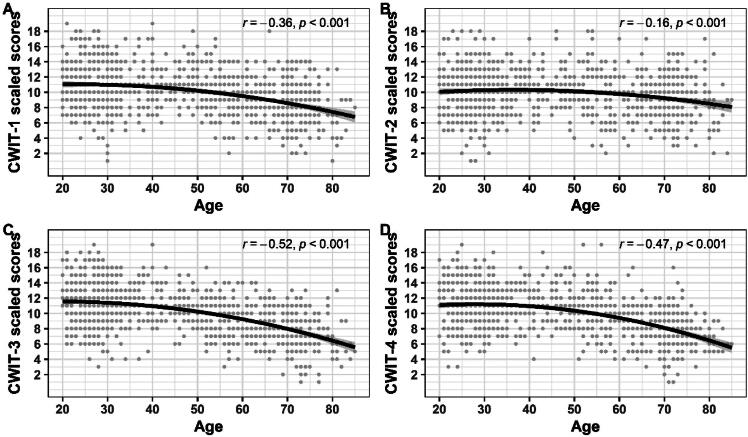
Scatterplots of age^2^ and CWIT 1-4 scaled scores in the normative sample (*n* = 1011).

**Figure A2.**
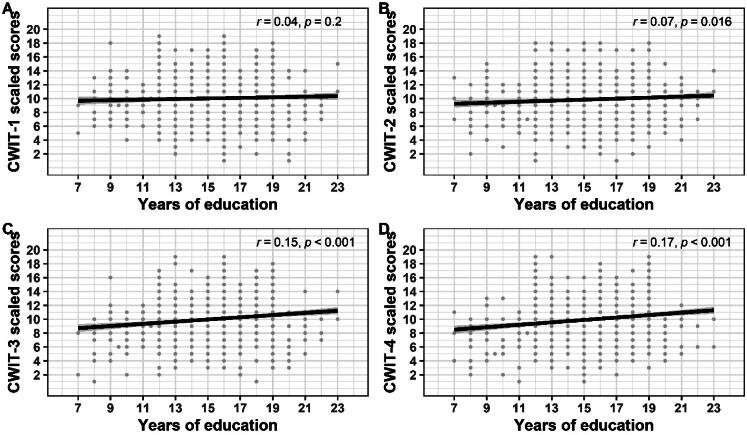
Scatterplots of years of education and CWIT 1-4 scaled scores in the normative sample (*n* = 1011).

We first conducted explorative analyses to evaluate CWIT outcomes and relations to demographic variables before fitting normative models. Pearson correlations indicated significant relationships between age, education, and sex with CWIT 1-4 time to completion (Table 2). We then assessed the distributions for each CWIT subtest for normality which indicated significant positive skewness and kurtosis due to slow completion times for a small part of the normative sample. To normalize measures, we transformed CWIT 1-4 outcomes to a scaled score distribution (*M* = 10, *SD* = 3) similar to Espenes et al. (2020), Kirsebom et al. (2019), and Testa et al. (2009). Measures were normalized using the package “CTT” in R (Willse, 2022). Raw scores were transformed to scaled scores by first determining the percentile ranks of raw scores on CWIT 1-4. Then, percentile ranks were converted to scaled scores in the reversed order so that higher scaled scores related to faster completion time. For instance, the 50^th^ percentile corresponds to scaled score 10, and the 99^th^ percentile corresponds to scaled score 17. Raw score to scaled score conversions are shown in Table 3. Univariate analyses showing the relationships between predictors age and years of education on CWIT 1-4 scaled scores are shown in appendix Figures A1 and A2.

**Table 2.** Pearson correlation between time to completion on CWIT 1-4 and demographical variables.

Parameter	Age	Age^2^	Education	Sex
CWIT-1 raw	.366*	.380*	−.033	−.187*
CWIT-2 raw	.124*	.135*	−.065	−.041
CWIT-3 raw	.500*	.519*	−.150*	−.134*
CWIT-4 raw	.450*	.470*	−.178*	−.084*
Errors CWIT-3^1^	−.068	−.068	−.051	.017
Errors CWIT-4^1^	−.014	−.014	−.042	.035

*Note*.

* Statistically significant (*p* <.01).

^1^ For errors, Spearman’s rho is reported for continuous variables. Sex-differences on errors were tested with Mann-Whitney test and the rank-biserial correlation is reported; CWIT = Color-Word Interference Test.

**Table 3.** Raw score to scaled score conversion on CWIT 1-4.

Scaled score	Percentile	CWIT-1	CWIT-2	CWIT-3	CWIT-4
1	0.1	≥56	≥52	≥130	≥168
2	0.4	48-55	37-51	114-129	133-167
3	1	46-47	34-36	99-113	117-132
4	2	42-45	31-33	85-98	101-116
5	5	40-41	28-30	77-84	88-100
6	9	37-39	27	69-76	78-87
7	16	35-36	25-26	63-68	71-77
8	25	33-34	24	58-62	65-70
9	37	31-32	23	53-57	60-64
10	50	29-30	21-22	49-52	55-59
11	63	27-28	20	45-48	51-54
12	75	26	19	42-44	48-50
13	84	25	18	40-41	45-47
14	91	23-24	17	37-39	42-44
15	95	22	16	35-36	39-41
16	98	21	15	33-34	37-38
17	99	20		31-32	34-36
18	99.6	19	≤14	27-30	31-33
19	99.9	≤18		≤26	≤30

*Note*. Scaled scores are not adjusted for demographical variables and are only used for computing the regression equations in Table 4; CWIT = Color-Word Interference Test.

To produce the regression-based norms we performed multiple regression analyses on the CWIT 1-4 scaled scores with age, education, and sex as predictors. We also assessed squared and cubic effects, and interaction terms. Education and age were centered around the mean (i.e. years of education − 15.5) and (age − 46.2) to avoid issues with multicollinearity. For the model selection process, we proceeded similarly to Van der Elst et al. (2006). We started with a full model including all terms related to performance on the CWIT subtests based on previous studies and explorative analyses (Table 2). The preliminary full model included age + age^2^ + age^3^ + sex + education + education^2^ + education^3^ + age*sex + education*sex + age*education. With the full model as a reference, we hierarchically dropped terms in a stepwise manner, and compared model fit with the simplified model. Models were compared with ANOVAs for total explained variance (*R*^2^), *p*value, and the Bayesian information criterion (BIC). The simplified model was preferred if *p* = ≥.01. The simplified model was subsequently used as reference for further simplification using the same alpha level criterion of α = .01. Regression models were reduced until the simplified model explained significantly less variance than the reference model (i.e. *p* = ≤.01). Lastly, we attempted to exchange squared terms in the final models with smooth functions using generalized additive models (GAMs). The model fit of the GAMs were compared to the linear models following the same procedure as described. BIC and ANOVAs favored the linear models with squared terms, and the smooth functions did not improve model fit to a substantial degree. After reaching the model structures with the best fit for CWIT 1-4 subtests (Table 4), we assessed assumptions of normality and heteroscedasticity using plots of standardized predicted scores and standardized residuals (James et al., 2021). Outliers and influential cases were visually assessed using plots of Cook’s distance and standardized residuals. Visual inspection revealed no markedly diverging observations, thus no observations were deleted based on statistical criteria. All analyses were conducted using R version 4.2.1 and packages “dplyr” (Wickham et al., 2022), “CTT” (Willse, 2022), “Psych” (Revelle, 2023) and “mgcv” (Wood & Wood, 2015).

**Table 4.** Normative regression models for CWIT (Color-Word Interference test) subtests 1-4 based on 1011 healthy Norwegian adults.

	Parameter	*b*	*b* 95 % CI [LL, UL]	*s.e.*	*t*	*p*	*Partial R^2^*	*Adj. R^2^*	*SD residual*
CWIT-1	Intercept	9.863	[9.474, 10.253]	0.20	49.71	<.001		.155	2.775
	Age	−0.049	[−0.059, −0.039]	0.01	−9.89	<.001	.088		
	Age^2^	−0.001	[−0.002, <−0.00]	<0.01	−3.12	.002	.010		
	Female	0.825	[0.456, 1.193]	0.19	4.39	<.001	.019		
CWIT-2	Intercept	10.217	[9.919, 10.515]	0.15	67.27	<.001		.031	2.797
	Age	−0.019	[−0.028, −0.009]	0.01	−3.76	<.001	.014		
	Age^2^	<-0.001	[−0.002, <−0.001]	<0.01	−2.80	.005	.008		
CWIT-3	Intercept	10.182	[9.824, 10.541]	0.18	55.67	<.001		.291	2.546
	Age	−0.073	[−0.081, −0.064]	0.01	−15.97	<.001	.202		
	Age^2^	−0.001	[−0.002, <−0.001]	<0.01	−3.89	<.001	.015		
	Edu	0.078	[0.022, 0.133]	0.03	2.75	.006	.008		
	Female	0.454	[0.116, 0.793]	0.18	2.64	.009	.007		
CWIT-4	Intercept	10.561	[10.284, 10.838]	0.14	74.84	<.001		.250	2.574
	Age	−0.063	[−0.072, −0.054]	0.01	−13.83	<.001	.160		
	Age^2^	−0.002	[−0.002, <0.001]	<0.01	−5.01	<.001	.024		
	Edu	0.098	[0.042, 0.154]	0.03	3.43	<.001	.012		

Note. *b* = unstandardized beta coefficient; s.e. = standard error of the unstandardized beta coefficient; SD residual = standard deviation of the residuals; Sex was coded 0 = men, 1 = women; Age and Education were mean centered, thus Age = (age - 46.2); Education = (the number of years of education obtained - 15.5); CWIT scores were transformed to scaled scores (*M* = 10, *SD* = 3) where higher scaled scores indicate increased test performance (Table 3).

#### Testing the equality of age coefficients on CWIT subtests

Adding to the regression analyses described previously, we considered if the effect of age significantly differed on CWIT subtests. For instance, while the effect of age might significantly predict scores on one subtest, and not the other, this does not infer that the effect of age is different on the subtests (Gelman & Stern, 2006). To test the equality of coefficients we fitted multivariate models (seemingly unrelated regressions) reproducing the normative analyses in Table 4 for two subtests at a time. Then, we tested whether the unstandardized beta coefficients from age obtained through this analysis were equivalent in both models using *Z*-tests (Table A1). For these analyses we used an alpha level criterion of α = .01 to reject the null hypothesis that the difference between the coefficients is zero (i.e. the coefficients are equal). Multivariate models were fitted because this allows for the calculation of standard errors that are adjusted for the covariance between beta coefficients. Analyses were conducted using R studio version 4.2.1 and the package “Systemfit” (Henningsen & Hamann, 2007) and *Z*-tests were conducted using the package “Multcomp” (Hothorn et al., 2016).

#### Errors on the Color-Word Interference Test

To provide normative estimates for errors on CWIT-3 and 4 we summarized corrected and uncorrected errors to a total error score. A total of 936 participants had data on errors. Unfortunately, as we did not record errors on CWIT-1 and 2, we do not provide data regarding the distribution of errors on these subtests. Preliminary analyses indicated that errors on CWIT-3 and 4 were zero-inflated and over-dispersed, as most participants did not make any errors during these subtests. Thus, the variables did not follow a normal distribution suitable for linear regression analysis. We conducted preliminary analyses to investigate if there were linear associations between errors on the CWIT-3 and 4 with age, education, and sex using Spearman’s ROH and Mann-Whitney U tests (Table 2). Analyses were done to assess the need for demographic adjustment or stratification for error measures. Results from these analyses indicated a weak association between errors on CWIT-3 and 4 with demographic variables. We therefore provide percentiles based on the inverse cumulative distribution for errors based on the entire normative sample unstratified according to demographic variables (i.e. unadjusted for age, education, or sex). We then dichotomized the sample into participants who performed 0 errors and ≥ 4 errors on either CWIT-3 or 4 to see if these groups might differ in years of education, age, or sex. In total, 14.1% of the sample made ≥ 4 errors on *either* the CWIT-3 or 4. Thus, ≥ 4 errors on either CWIT-3 or 4 corresponded to a “low average” score according to neuropsychological nomenclature (Guilmette et al., 2020). We then assessed whether errors on CWIT-3 and 4 were related to performance on the task. First, we compared completion time on CWIT-3 and 4 between individuals who made ≥ 4 errors and individuals who made 0 errors using two-tailed independent samples t-tests without assumptions of equal variance. Further, we correlated errors on CWIT-3 and 4 with time to completion on CWIT-3 and 4 to check for a linear relationship between errors and task performance.

#### Calculating normative performance using regression-based norms

To determine the normative performance for a given individual (i) on a given test (j), we first calculate the predicted scaled score using the regression equations presented in Table 4. These equations utilize the following formula: Let D be a set of demographic predictors, where d_n_ represents the n-th element of D; Predicted scaled score_ij_ = intercept_j_ + sum(beta_coefficient_dj_ * d_ni_). Then, the individual’s raw score on the CWIT is converted to a scaled score using the raw score to scaled score conversion in Table 3. This reflects the individual’s obtained scaled score. Lastly, the *Z*-score of individual (i) on test (j) is computed by [*Z*_ij_ = (obtained scaled score_ij_—predicted score_ij_)/standard deviation of the residual_j_], which can be further converted to a *T*-score by [(Z_ij_*10) + 50].

#### Assessing established American norms from D-KEFS in the Norwegian sample

**Figure 1.**
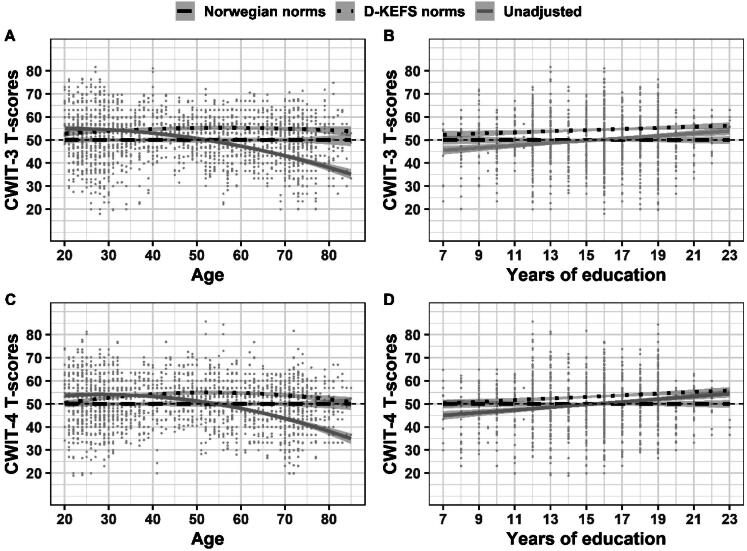
Plots Of T-scores on CWIT-3 and CWIT-4 calculated applying norms from D-KEFS, Norwegian norms, and T-scores unadjusted for demographic variables. *Note*. Linear regression lines are fitted for years of education and squared lines for age; for all figures a horizontal line from *T* = 50 represents the ideal normative correction and deviation from this line may indicate maladjustment in the norms. CWIT = Color-Word Interference Test. D-KEFS = Delis-Kaplan Executive Function System.

**Figure 2.**
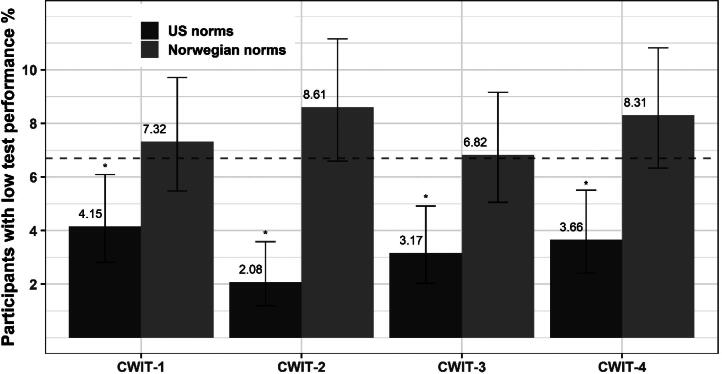
Percentage of participants in the Norwegian sample (*n* = 1011) with a score 1.5 SD below the normative mean (T-score < 35) on CWIT (Color-Word Interference Test) subtests 1-4. *Note*. Dotted line indicates the expected base rate for 1.5 *SD* below the normative mean (6.7%). Error bars indicate the 99% confidence interval (*CI*) around the estimate. *CI does not contain the expected base rate (*p* <.01). Paired samples proportion tests indicated significant difference between rates from US norms and Norwegian norms on all CWIT subtests (*p* <.001).

**Figure A3.**
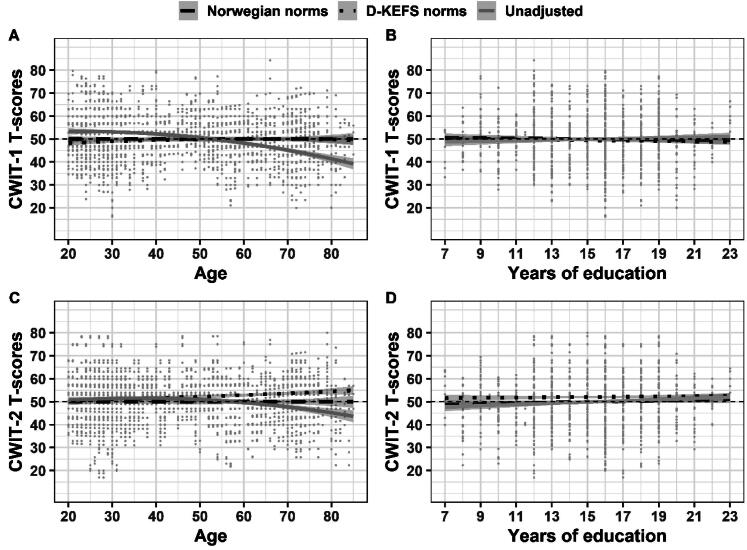
Plots of T-scores on CWIT-1 and CWIT-2 calculated applying norms from D-KEFS, Norwegian norms and T-scores unadjusted for demographic variables.

We computed *T*-scores based on the original age-adjusted norms from the D-KEFS manual (Delis et al., 2001) on CWIT 1-4 for all participants (*n* = 1011). This resulted in four *T*-scores for each participant; *T*-score on the CWIT-1; CWIT-2; CWIT-3; CWIT-4. To assess if the original age-adjusted norms from D-KEFS sufficiently adjusted for demographical variables in the Norwegian sample, we performed multiple regression analyses with CWIT 1-4 *T*-scores as dependent variables. Age, years of education, and sex were used as predictors for all analyses. A significant beta-coefficient from any predictor was interpreted as a mal-adjustment in the norms. For these analyses we used a conventional alpha level criterion of α = .05. For example, if years of education significantly explained variance in the *T*-scores, this was interpreted as if the norms did not adequately correct for this demographic variable. Non-significant results were interpreted as an adequate adjustment. *T*-scores using the new Norwegian norms were calculated for all participants following the procedures detailed in the previous section. We then compared mean *T*-scores for all participants on the CWIT 1-4 using both the norms from D-KEFS and the new Norwegian norms. Mean *T*-scores on the CWIT 1-4 were compared using paired samples *T*-tests without the assumption of equal variances (Table 7). Plots of *T*-scores on CWIT-3 and CWIT-4 with fitted regression lines for the new Norwegian norms, the D-KEFS norms, and unadjusted *T*-scores are compared in Figure 1. Corresponding plots of *T*-scores on CWIT-1 and CWIT-2 are included in appendix Figure A3. Lastly, we compared the observed rate of participants scoring below a conventional cut-off (1.5 *SD* below the normative mean; *T*-score < 35) on CWIT 1-4 applying the original age-adjusted norms from D-KEFS and the Norwegian norms. Because the *T*-scores are expected to approximate a normal distribution we used two-tailed one proportion *Z*-tests to compare the observed rate in the samples with the expected base rate in a theoretical normal distribution (6.7%). The *Z*-test estimates the probability that the observed sample proportion is equal to the theoretical proportion in the population. For these tests we computed the 99% confidence interval around the sample proportion thereby using a significance level of α = .01 (Figure 2). To test if there were significant differences in proportions between the Norwegian norms and the original age-adjusted D-KEFS norms we used paired-samples proportion tests (asymptotic McNemar test without Continuity Correction) (Fagerland et al., 2014).

**Table 7.** Paired sample t-tests between T-scores computed using the Norwegian norms and original age-adjusted norms from D-KEFS.

	*M (SD)*	*t*	*p*	*M*diff	95% CI *M*diff	Cohen’s d
D-KEFS norms CWIT-1	49.5 (7.7)	−5.085	<.001	−0.55	[-0.76, −0.34]	−0.16
D-KEFS norms CWIT-2	52.1 (7.3)	16.055	<.001	2.06	[1.83, 2.30]	0.54
D-KEFS norms CWIT-3	54.4 (8.4)	38.267	<.001	4.41	[4.19, 4.64]	1.20
D-KEFS norms CWIT-4	53.2 (8.5)	25.436	<.001	3.22	[2.97, 3.47]	0.80

*Note*. Df = 1010; *T*-scores computed using Delis et al. (2001) norms were always paired with Norwegian norms that had a mean of 50 (*SD* = 10); CWIT = Color-Word Interference Test.

#### Norm calculator

To make regression-norms available and easy to use, we provide a free web-based tool that computes the regression equations and provide demographically adjusted *T*-scores for all CWIT subtests. The tool will be implemented as a self-contained HTML/Javascript webpage but is temporarily available at (https://contattafiles.s3.us-west-1.amazonaws.com/tnt30503/ACkqU46CjUb0rss/cwit-calc.html) and is released as open source at (https://github.com/DDI-NO/cwit-calc) under Apache License, version 2.0.

#### Stability over time on the CWIT

A sub-set of the normative sample (*n* = 335) had available follow-up assessments allowing for test-retest correlations assessing stability over time. The sample consisted of 207 women (62%) and 128 men (38%) with a mean age of 52.6 years (*SD* = 18.4) and 15.6 (*SD* = 2.9) years of education at baseline. To ensure that stability indexes remained unified and relevant for clinical practice, participants tested later than 5 years after follow-up were excluded from the analysis (*n* = 22). Thus, the average time between assessments varied between 1 and 5 years with an average test-retest interval of 3.4 years (*SD* = 0.9). Intraclass correlation (ICC) estimates and 95% CIs were calculated based on a single rating, absolute-agreement two-way mixed-effects model (Shrout & Fleiss, 1979). We specified *a priori* ranges for stability based on conventional reliability classifications from (Koo & Li, 2016). Values between 0.5 − 0.75 indicate moderate stability and 0.75–0.9 indicate good stability. To determine whether the difference in score on a CWIT subtest between baseline and follow-up obtained by an individual represent a significant difference, the score may be analyzed considering the Reliable Change Index (RCI). In RCIs, the difference score is divided by the Standard Error of Measurement of the Difference thus providing a standardized *Z*-score describing whether the change in score between baseline and follow is statistically significant (i.e. represent a reliable change) (Guhn et al., 2014). In Appendix Table A2, we provide readers with the necessary statistics to calculate RCIs themselves *via* the most common methods. For a review, please refer to Guhn et al. (2014).

### Ethics

The Norwegian Regional committees for medical and health research ethics (REK) approved the projects the current study draws upon. Guidelines in the Helsinki declaration of 1964 (revised 2013) and the Norwegian Health and Research Act were followed. All participants gave written informed consents, and were informed of their right to withdraw, as well as potential risks and rewards involved with participation.

## Results

### Effect of age, education, and sex on CWIT performance

Higher age was on average related to worse performance on all CWIT measures (Table 4). The effects of age and age^2^ were strongly related to performance on CWIT-1 (*b* = −0.049, partial *R*^2^ = 8.8%, *p* = <.001) (*b* = −0.001, *p* = .002, partial *R*^2^ = 0.10%), CWIT-3 (*b* = −0.073, *p* = <.001, partial *R*^2^ = 20.2%) (*b* = −0.001, *p* = <.001, partial *R*^2^ = 1.5%), and CWIT-4 (*b* = −0.063, *p* = <.001, partial *R*^2^ = 16%) (*b* = −0.002, *p* = <.001, partial *R*^2^ = 2.4%). However, on CWIT-2, the association with age and age^2^ was weaker (*b* = −0.019, *p* = <.001, partial *R*^2^ = 1.4%) (*b* = <-0.001, *p* = .005, partial *R*^2^ = 0.8%). Tests of the equality of coefficients indicated that the effect of age was stronger on the complex trials CWIT-3 and CWIT-4 compared to CWIT-1 and CWIT-2 (Table A1). Furthermore, the effect of age was significantly weaker on CWIT-2 compared to all other subtests (*p* = <.001). Figure A1 shows the linear and quadratic effect of age on all CWIT subtests in the normative sample between 20 and 85 years.

There was a weak but significant positive relationship between years of education and scores on CWIT-3 (*b* = 0.078, *p* = .006, partial *R*^2^ = 0.8%) and CWIT-4 (*b* = 0.098, *p* = <.001, partial *R*^2^ = 1.2%). However, there were no significant associations between years of education and performance on the basic tasks CWIT-1 and CWIT-2 adjusted for sex and age. The relationship between CWIT scores and years of education is shown in Figure A2.

Women performed significantly better than men on CWIT-1 (*b* = 0.825, *p* = <.001, partial *R*^2^ = 1.9%) and CWIT-3 (*b* = 0.454, *p* = .009, partial *R*^2^ = 0.7%). On average, women attained 0.83 higher scaled scores on CWIT-1, and 0.45 on CWIT-3 (Table 4). There were no significant interactions between sex and age, sex and education, or age and education for any CWIT subtests.

#### Calculating normative performance on CWIT-1 using regression-based norms

As an example, suppose that a 70-year-old man with 17 years of education completed the CWIT-1 in 35 s. The mean age in the normative group was 46.2 and the mean years of education was 15.5 (Table 1). First, we obtain the relevant coefficients from Table 4. The predicted scaled score is calculated by [9.863 + ((70 − 46.2) *(-0.049)) + ((70 − 46.2)^2^ * −0.001) + (0 * 0.825)] which is 8.13. From Table 3 we see that a 35 s completion-time on CWIT-1 equates to a scaled score of 7. Thus, the demographically adjusted Z-score is calculated by [(7 − 8.13)/2.775] giving a Z-score of −0.41. The Z-score can be further converted to a *T*-score with a mean of 50 and standard deviation of 10 by [(-0.41*10) + 50] = *T* 46.

### Errors on CWIT-3 and CWIT-4

As shown in Table 2, there were no significant linear associations between demographic variables and errors on CWIT-3 or CWIT-4. Due to the weak association with demographic variables, we report the cumulative percentiles associated with number of errors based on a subset of the normative sample (*n* = 936) unstratified for age, sex, or educational attainment (Table 5).

**Table 5.** Total errors on CWIT-3 and CWIT-4 in a subset of the normative sample (*n* = 935).

	Cumulative percentages	
Errors	CWIT-3	CWIT-4
0	100	100
1	51.4	55.4
2	23.2	26.5
3	11.1	12.9
4	5.2	6.3
5	2.8	3.2
6	1.2	1.5
7	0.5	1.1
8	0.4	0.6
9	0.2	
10		
11	0.1	0.2

*Note*. Cumulative percentages show proportion of the normative sample that attained *k* number of errors (or more); CWIT = Color-Word Interference Test.

More errors on the CWIT were associated with longer time to completion on the CWIT. Pearson correlations indicated a positive linear association between total number of errors on CWIT-3 and time to completion, *r*(935) = .28, 95% CI [.219, .338], *p* = <.001, and total errors on the CWIT-4 and time to completion, *r*(935) = .408, 95% CI [.353, .460], *p* = <.001). To illustrate, we dichotomized participants into two groups with ≥4 errors on either CWIT-3 and CWIT-4 indicating a “low average” score (*n* = 41), and participants with 0 errors (*n* = 250). As expected, there were no significant differences between groups in age, years of education, or sex. However, participants who made ≥ 4 errors on either task completed the CWIT-3 9.2 s slower (*M* = 58.7, *SD* = 18.9) compared to participants with no errors (*M* = 49.5, *SD* = 12.2), *t*(45.6) = −3.01, *M*_diff_ = −9.2, *p* = .004. On the CWIT-4, participants with ≥ 4 errors completed the subtest 24.4 s slower (*M* = 79.7, *SD* = 28.9) compared to the group with 0 errors (*M* = 55.2, *SD* = 13.6), *t*(43.0) = −5.32, *M*_diff_ = −24.4, *p* = <.001.

### Assessing established norms from D-KEFS in the Norwegian sample

As shown in Table 6, results from multiple regression analysis on *T*-scores calculated using the original age-adjusted D-KEFS norms indicated significant positive effects of age on all CWIT trials, meaning higher age predicted higher *T*-scores. As shown previously, women performed better than men on CWIT-1 and 3 in the Norwegian sample. However, the norms from D-KEFS did not account for this sex difference, and on average, women attained 2.3 and 1.4 higher *T*-scores compared to men on the CWIT-1 and 3 (Table 6). Moreover, there was a significant positive association between years of education and CWIT-3 and CWIT-4 *T*-scores, where participants with higher levels of education received higher *T*-scores. The combined effect of demographic variables in the age-adjusted scores were low, ranging from 1.6% to 3.0% explained variance. Nevertheless, there were significant mean differences between the D-KEFS norms and the new Norwegian norms (Table 7). On all trials except CWIT-1, the D-KEFS norms produced too high *T*-scores compared to the expected mean value of *T* = 50. On CWIT-2 the average *T*-score using the D-KEFS norms was 52.1; *T =* 54.4 on CWIT-3; and *T =* 53.2 on the CWIT-4.

**Table 6.** Results from multiple regression analysis on T-scores calculated with the original D-KEFS norms in the normative group (*n* = 1011).

		Original D-KEFS norms			
Variable	Predictor	*b*	*p*	Partial *R*^2^	Adj. *R*^2^
CWIT-1	Intercept	47.913	<.001		.023
	Age	0.037	<.004	.008	
	Education	−0.073	.380	.001	
	Sex	2.308	<.001	.020	
CWIT-2	Intercept	51.767	<.001		.030
	Age	0.069	<.001	.032	
	Education	0.102	.195	.002	
	Sex	0.442	.362	.001	
CWIT-3	Intercept	53.465	<.001		.016
	Age	0.039	.005	.008	
	Education	0.263	.004	.008	
	Sex	1.418	.012	.006	
CWIT-4	Intercept	52.710	<.001		.023
	Age	0.047	<.001	.011	
	Education	0.382	<.001	.017	
	Sex	0.762	.177	.002	

*Note. b* = unstandardized regression coefficient; *p* =* p*value; partial *R*^2^ = explained variance of predictor variable; Adj. *R*^2 =^ explained variance of combined predictor variables; significant coefficients (*p* >.05) indicate mal-adjustment in the norms; Age and education was mean centered; CWIT = Color-Word Interference Test.

When utilizing the original age-adjusted norms from D-KEFS the proportion of participants scoring 1.5 *SD* or more below the normative mean was significantly different compared to the expected base rate on all CWIT subtests (Figure 2). The Norwegian norms were not significantly different compared to the expected base rate and the 99% *CI*s contained the expected base rate for all subtests (*p* >.01). Results from paired samples proportion tests showed significant differences between the estimated proportion of participants with scores 1.5 *SD* or more below the normative mean using the Norwegian norms or the original age-adjusted D-KEFS norms (*p* <.001) (Figure 2).

### Stability over time on the CWIT

Intra-class correlation coefficients and 95% CIs are shown in Table 8. Based on the *a priori* specified ranges, all analyses indicated moderate to good stability in scores between baseline and follow-up using the Norwegian CWIT norms. Slightly lower estimates were obtained with the original D-KEFS norms.

**Table 8.** Intra-class correlations between baseline and follow-up on D-KEFS CWIT subtests based on a sub-set of the normative sample (*n* = 335).

Measure	ICC	95% CI [LL, UL]
CWIT-1 D-KEFS norms	.69	[.63, .74]
CWIT-1 Norwegian norms	.68	[.62, .74]
CWIT-2 D-KEFS norms	.62	[.55, .68]
CWIT-2 Norwegian norms	.68	[.61, .73]
CWIT-3 D-KEFS norms	.73	[.67, .78]
CWIT-3 Norwegian norms	.76	[.71, .80]
CWIT-4 D-KEFS norms	.66	[.59, .71]
CWIT-4 Norwegian norms	.70	[.64, .75]

*Note*. ICC = intraclass correlation coefficient; ICC based on single rating, absolute-agreement two-way mixed-effects model; CWIT = Color-Word Interference Test; D-KEFS = Delis-Kaplan Executive Function System.

## Discussion

### Effects of demographics on the D-KEFS CWIT

We present normative data for the D-KEFS CWIT based on the performance of 1011 healthy Norwegians between 20 and 85 years of age. All four CWIT test scores were related to linear and quadratic effects of age, indicating a steepening trend towards lower scores for older participants. Quadratic effects of age have been reported on Stroop tests in similar samples spanning the entire adult range (Ktaiche et al., 2022; Van der Elst et al., 2006), but rarely in samples with more restrictive age spans (Bayard et al., 2011; Bezdicek et al., 2015; Magnusdottir et al., 2021; Seo et al., 2008; Tremblay et al., 2016). Consistent with most studies, we found that the basic subtests CWIT-1 (color naming) and CWIT-2 (word reading) were significantly less influenced by age compared to the complex inhibition trial (CWIT-3) and the inhibition/switching trial (CWIT-4) (Adólfsdóttir et al., 2014; Mitrushina et al., 2005).

Scores on the CWIT may decline with age due to a general age-related slowing of information processing (Salthouse, 1996) and specific deficits in executive functions like inhibitory control (Hasher & Zacks, 1988). Indeed, Adólfsdóttir et al. (2017) showed that higher age significantly predicted slower time to completion on CWIT-3 and 4 after adjusting for processing speed and performance on CWIT-1 and CWIT-2. In other words, when basic non-executive functions were regressed out, there was still an age effect on both CWIT-3 and CWIT-4, thereby implying that there was a specific factor associated with aging beyond generalized slowing. Delis et al. (2001) published contrast measures in the original D-KEFS norms to isolate executive components on the CWIT. However, these contrasts rely on simple subtraction between individual subtest scores, and it has been suggested that this approach might multiply the measurement errors on each test leading to low reliability (Crawford et al., 2008). Unpublished data from the same Norwegian sample used in this study support this, and we hypothesize that a regression-based approach to isolate executive components could mitigate this problem. We therefore aim to develop norms on CWIT-3 and CWIT-4 adjusted for performance for basic tasks using a regression-based approach and compare test-retest reliability with the original contrast scores from D-KEFS in a separate paper.

#### Effects of education on CWIT scores

Education was significantly, albeit weakly associated with scores on the CWIT-3 and CWIT-4 but was not significantly associated with scores on CWIT-1 and CWIT-2. This is in line with previous studies, where education has been reported to exert a strong influence on the complex Stroop inhibition trial (Bayard et al., 2011; Bezdicek et al., 2015; Brugnolo et al., 2016; Ktaiche et al., 2022; Magnusdottir et al., 2021; Van der Elst et al., 2006). Education is positively associated with full scale IQ (Ritchie & Tucker-Drob, 2018; Steinberg et al., 2005) which might explain why education was related to performance on the complex trials specifically. Moreover, cognitive reserve (Stern et al., 2023) has commonly been proposed as an explanation for how education is related to scores on Stroop tests (Hankee et al., 2016; Ktaiche et al., 2022; Seo et al., 2008; Zalonis et al., 2009). Relating to Stroop tests, Van der Elst et al. (2006) showed that individuals with low educational attainment had an accelerated lowering of performance with age compared to individuals with an average or high level of education. This indicates that the individuals with more education were resilient to age-related brain changes and pathology. However, our results indicated a positive effect of education on CWIT-3 and CWIT-4 scores that was independent of age. Therefore, our results are unlikely to be related to increased cognitive reserve.

Compared with our results, some studies report stronger associations between performance on Stroop tests and education (Hankee et al., 2016; Magnusdottir et al., 2021) while others report comparable associations (Bayard et al., 2011; Troyer et al., 2006). The weak relationship between education and CWIT scores observed in our study might be influenced by sample characteristics in the normative sample. In particular, the Norwegian sample comprised individuals with relatively uniform and high educational attainment (*M* = 15.5, *SD* = 2.9). So, it follows that samples with uniform levels of education have reduced variance explained by educational attainment. Furthermore, some studies have indicated that the effect of education on scores could be less impactful for the highly educated (Van der Elst et al., 2006), and that the effect of education on Stroop performance could be diminishing after approximately 12 years (Hankee et al., 2016). Reports from Statistics Norway indicate that the educational level of the adult population is divided into three approximately equal parts (Statistics Norway, 2022); mandatory schooling (10 years education); high school level including trade schools (≤13 years); university degrees of various lengths (14+ years). Thus, the sample in this study had higher educational attainment than the population average, which may have influenced the relatively weak effect of education on CWIT scores. However, the education range in our sample was 7 to 23 years, and pertinent educational effects on test performance are modelled in our norms at both lower and higher levels of education. The discrepancy between norms is difficult to pinpoint as it could be influenced by several other factors, including the normative estimation method and a variety of cultural influences like educational quality and availability. Regardless, differences between norms highlight the importance that the normative sample resemble the intended population in terms of sample characteristics and geography (Heaton et al., 1999; International Test Commision, 2001). Specifically, using estimates from foreign samples exhibiting strong effects of education (e.g. Peña-Casanova et al., 2009; Seo et al., 2008) would likely provide inaccurate estimates of performance in the Norwegian sample where education evidently is not as relevant for predicting performance on the CWIT.

#### Sex differences

Women performed significantly better than men on CWIT-1 (color-naming) and CWIT-3 (inhibition). Previous studies on various Stroop paradigms report inconsistent results regarding sex differences with some studies reporting significant sex-differences (Magnusdottir et al., 2021; Mitrushina et al., 2005; Seo et al., 2008; Tremblay et al., 2016; Van der Elst et al., 2006) while others do not (Adólfsdóttir et al., 2017; Bayard et al., 2011; Hankee et al., 2016; Zalonis et al., 2009). Despite this, any observed difference has consistently favored women. Therefore, it is likely that the effect is small and that a large sample size is needed to detect a sex difference on Stroop tests. A recent article by Sjoberg et al. (2023) proposed that the female advantage on Stroop paradigms is related to superior color-naming abilities likely attributed to several specific verbal abilities relevant to performance on the task. These include increased speed on color labelling tasks and better performance on distractor suppression tasks. For a full review, please see Sjoberg et al. (2023). This could explain why we only found a female advantage on CWIT-1 (color naming) and CWIT-3 (inhibition), which has more color stimuli than CWIT-2 (word reading) and CWIT-4 (inhibition/switching).

#### Errors on CWIT-3 and CWIT-4

Number of errors on the CWIT were not related to age, education, or sex, which is surprising considering existing literature that report significant effects (Tremblay et al., 2016; Troyer et al., 2006; Van der Elst et al., 2006; Zalonis et al., 2009). Hankee et al. (2016) report that participants who made errors were significantly older and had less education compared to those with 0 errors. The present study did not find demographic differences between individuals with ≥4 errors compared to those with 0 errors. However, consistent with previous studies, our results indicate that errors were significantly related to worse performance on the task. On average, errors on the CWIT-3 and CWIT-4 were correlated with increased time to completion, and participants with ≥4 errors completed the CWIT-3 and CWIT-4 significantly slower. For clinical decision making, ≤ 3 errors on CWIT-3 and CWIT-4 should be considered the lower boundary for normal performance corresponding to the ∼11–13^th^ percentile (Table 5). Unfortunately, we do not provide normative estimates for errors on CWIT-1 and CWIT-2. Previous studies indicate that about one in 20 healthy participants make one error on the CWIT-1 or CWIT-2 (Bayard et al., 2011) and multiple errors on these subtests may therefore indicate issues concerning the validity of the test performance. For normative estimates on CWIT-1 and 2 we refer to the original D-KEFS norms by Delis et al. (2001).

### Assessment of the original age-adjusted norms from D-KEFS and clinical implications

A key aim of this study was to assess the adequacy of the original age-adjusted norms from D-KEFS in our sample of healthy Norwegians (*n* = 1011). Higher age significantly predicted higher *T*-scores calculated using norms from D-KEFS. From Figure 1 we can see that the yellow line is above the reference line for *T* = 50 which means that the participants on average performed better than the normative mean from D-KEFS given their age. This indicates that the original norms from D-KEFS slightly exaggerated the detrimental effects of aging on CWIT performance in the Norwegian sample. As a result, the older participants in the Norwegian sample received slightly elevated *T*-scores on average. Previous studies have found that age-related decline on cognitive tests largely dissipate when adjusting for cerebrovascular pathology, degeneration of structural and functional brain connections, and other pathologies (Anders M. Fjell et al., 2017; Borghesani et al., 2013; Borland et al., 2020; Harrington et al., 2018; Yu et al., 2015). Age-related decline on the CWIT could therefore be influenced by sub-clinical pathology. Notably, such sub-clinical pathology may be regarded as normal, since most studies with normal healthy participants screened for various pathological conditions indeed report a strong influence of age on scores from Stroop paradigms and other neuropsychological tests (Mitrushina et al., 2005). However, the extent may vary between cohorts. As a result, the comparatively weaker age-effect observed in the Norwegian sample could be due to the Norwegian sample being healthier. These potential differences could be cultural, such as differences in lifestyle and access to health care, or simply cohort-specific, such as cerebrovascular disease prevalence in the study sample. For instance, the Norwegian sample consisted of predominately highly educated individuals that were thoroughly screened which may have caused an over-representation of protective factors in the sample.

The difference between norms may not only be due to cultural differences as cohort differences are observed within cultures as well (Trahan et al., 2014). While data regarding Stroop tests specifically is scarce, the literature on other cognitive tests suggests that average cognitive functioning in today’s elderly is improved compared to the elderly 20 years ago (Hessel et al., 2018; Skirbekk et al., 2013). For younger individuals it is less clear with some studies showing that today’s young may perform similarly or worse (Bratsberg & Rogeberg, 2018). The improvement of newer cohorts over older cohorts is called the Flynn-effect, which stipulates that improvements in nutrition, educational attainment and quality, health care, health promoting activities such as exercise, and reduction in cardiovascular disease cause newer cohorts to perform better on a variety of cognitive task (Skirbekk et al., 2013). Thus, the disparity between the Norwegian norms and the original age-adjusted norms from D-KEFS published in 2001 may also be due to time of measurement.

Unsurprisingly, the original age-adjusted norms from D-KEFS did not account for the difference between individuals with high or low educational attainment or the female advantage we observed in the Norwegian sample. As a result, the norms from D-KEFS on average produced higher than expected *T*-scores on the CWIT-2 (2.1 *T*-scores), CWIT-3 (4.4 *T*-scores), and the CWIT-4 (3.2 *T*-scores™) compared to the expected value of *T* = 50. As shown in Figure 1, the difference between norms is most apparent for individuals in the midlife and/or individuals with high educational attainment. From a clinical point of view, using the D-KEFS norms in Norwegian samples could have implications for the accurate assessment of individuals with either very high or very low educational attainment, especially those in the midlife. To illustrate, an 80-year-old woman enrolled in this study reported 17 years of education and performed the CWIT-3 in 78 s and the CWIT-4 in 85 s. According to the Norwegian norms, her scores equate to *T* = 43 on CWIT-3, and *T* = 47 on CWIT-4, thus reflecting a below average performance. Using the D-KEFS norms her scores were *T* = 57 on both tasks, i.e. 1.4 *SD* and 1 *SD* higher compared to the Norwegian norms. For individuals with educational attainment or age closer to the sample average the choice of norms will on average yield less dissimilar results, but depending on the raw score of the individual the difference between the norms could still cause meaningful differences. For instance, using our Norwegian norms, a 55-year-old woman with 12 years of education with the same raw scores as in the previous example is estimated a score of *T* = 32 on CWIT-3 and *T* = 36 on CWIT-4. In comparison, the D-KEFS norms estimate her scores to *T* = 40 and *T* = 43, respectively. As a result, the difference between the Norwegian norms and the D-KEFS norms (0.8 *SD* and 0.7 *SD*, respectively) is smaller compared to the previous example. However, the Norwegian norms indicate a score on CWIT-3 more than −1.5 *SD* below the normative mean indicative of a potential deficit, while the D-KEFS norms indicate a score merely below average (-1 *SD* below the normative mean). Thus, while the average difference between the norms was estimated to *T* = 4.4 on CWIT-3 and *T* = 3.2 on CWIT-4, the difference might vary more depending on the age and/or years of education of the individual. Furthermore, depending on the obtained raw score of the individual these differences might lead to differences in diagnosis, however the diagnostic accuracy of the norms needs to be validated in future studies using independent samples.

We found that the proportion of participants scoring below a conventional cut-off set at 1.5 *SD* below the normative mean significantly differed from the expected proportion when using the original age-adjusted norms from D-KEFS. From Figure 2 it is apparent that the D-KEFS norms located fewer-than-expected participants with low scores on all CWIT subtests. Furthermore, the percentage of participants with low scores significantly differed between the norms with the D-KEFS norms identifying significantly fewer participants (*p* <.001). This indicates that the norms from D-KEFS have a lower sensitivity for identifying individuals with low scores on the CWIT in the Norwegian sample which might have important clinical implications. Although not statistically significant, the Norwegian norms located more participants with low scores on CWIT-2 and CWIT-4 than we expected (8.6% and 8.3% respectively). The Norwegian norms were expected to match the theoretical base rate of 6.7% more closely since the norms were produced in the same sample and scores were transformed to follow a normal distribution. The difference is likely caused by some skewness in the CWIT-2 and 4 scaled scores despite the normalization procedures which caused a slight over representation of participants around this cut-off. Future studies should assess the Norwegians norms in an independent sample of Norwegians to address whether the new norms equal the theoretical base rate of impairment, and preferably investigate the diagnostic accuracy of the norms for diagnosing MCI in samples including patients with confirmed MCI.

#### Correlations between baseline performance and follow-up on the CWIT

All psychological tests should have available evidence of reliability that is relevant to the intended population (International Test Commission, 2001). Ryder (2021) identified that tests from the D-KEFS battery were lacking reliability estimates based on a Norwegian sample. In this study we had test-retest scores based on a relatively long follow-up (*M* = 3.4 years), and test-retest correlations are therefore assumed to not just be a measure of reliability, but also reflect true change rates with age. For instance, a low correlation would typically be interpreted as low reliability, but it could also mean that some participants have a different slope (i.e. change rate) from baseline to follow-up. We therefore characterize the test-retest correlations as stability of scores over time. A limitation concerning these analyses is that the cognitive status of participants was not assessed at follow-up examinations, and it is therefore possible that some participants worsened in their cognitive status between baseline and follow-up. This would likely cause lower ICCs, meaning that our estimates might underestimate the stability of scores over time. However, as seen in Table A2, the mean scores between baseline and follow-up were on average very similar and it is doubtful whether this had large influences on estimates. The results from reliability analysis indicated moderate to good correlations for all measures, with slightly better correlation for the complex trials CWIT-3 and CWIT-4 (Table 8). Using the Norwegian norms resulted in marginally better correlation compared to the original D-KEFS norms, likely due to the slight mal-adjustments in age, education, and sex previously reported. The difference between coefficients using Norwegians norms and D-KEFS norms were not tested, although the 95% *CI* overlapped and the difference in coefficients would likely not fulfill conventional criteria for statistical significance.

## Limitations

The current study is subject to some limitations. Firstly, neuropsychological norms are typically intended to give an estimate of an individual’s score compared to a broad target population, e.g. healthy Norwegians between 20 and 85 years old. The representativeness of a normative sample is therefore crucial for the accuracy of the normative estimates. Most of the participants included in this study were recruited as healthy participants from advertisements, university, and workplaces, and could be susceptible to biases associated with convenience sampling methods. That is, the sample estimates may not generalize to the broad target population due to unknown biases arising from a non-probability sampling method (Jager et al., 2017). Relatedly, the sample was composed of native Norwegian speakers predominantly of European ethnicity which does not reflect the multicultural landscape in Norway. Unfortunately, we did not record the ethnoracial background of participants, however most were likely of European ethnicity. As a result, the norms are likely less accurate for people with Norwegian as a second language and immigrants to Norway. Despite this, as the first normative study outside the original age-adjusted norms presented in the D-KEFS manual (Delis et al., 2001) we believe our norms contribute to an improvement in the accuracy of CWIT assessments in Norway. Another limitation of this study is the lack of participants in the middle-age. However, the norms rely on the joint estimation of the average effect across the included age span to calculate predicted scores and deviation from the predicted scores. Thus, it is unlikely that the lack of participants in the middle-age greatly affect the norms’ ability to predict scores for individuals in this age range. Unfortunately, we were not able to source an independent sample to compare the new Norwegian norms with the original age-adjusted norms from D-KEFS. Instead, we assessed the adequacy of the original D-KEFS norms in our normative sample. Future studies should assess the validity of both the new norms and the original D-KEFS norms in an independent sample of Norwegians. Lastly, we did not formally screen for visual impairment or color blindness in participants but relied on self-report of visual deficits. Though participants were encouraged to use glasses when applicable, we cannot guarantee that undiagnosed visual impairment did not influence some participants’ scores.

## Conclusion

We propose regression-based norms for the Delis-Kaplan Color Word Interference Test (CWIT) based on a sample of healthy Norwegian adults between 20 and 85 years old (*n* = 1011). As far as we know, this is the first published study providing norms on the D-KEFS CWIT apart from the original age-adjusted norms from D-KEFS (Delis et al., 2001). Our results indicate that lower age, higher education, and female sex significantly predicted improved performance on the CWIT. The original age-adjusted norms from D-KEFS on average overestimated the difference between young and old participants and did not adjust for the female advantage or effects of education in the Norwegian sample. Consequently, normative estimates from the original D-KEFS norms may be inaccurate individuals with either low or high educational attainment, especially those in the midlife. The norms from D-KEFS identified significantly fewer-than-expected participants with low scores on CWIT 1-4 in the Norwegian sample. Low scores were defined as scores 1.5 *SD* or more below the normative mean. Thus, the D-KEFS norms had a lower sensitivity for detecting individuals with potential executive deficits compared to the Norwegian norms. In the Norwegian sample, ≥4 errors on CWIT-3 and CWIT-4 corresponded to the ∼5^th^ percentile, indicative of a borderline impaired performance. Errors were unrelated to demographical variables, but increased number of errors were significantly related to slower time to completion on the CWIT-3 and CWIT-4. The CWIT showed moderate to good test-retest stability in the Norwegian sample with a 3.4-year average follow-up time. For ease of use and quick computation of the norms we provide a normative calculator available at (https://contattafiles.s3.us-west-1.amazonaws.com/tnt30503/ACkqU46CjUb0rss/cwit-calc.html).

## Data Availability

The data that support the findings of this study are available on request from the corresponding author. The data are not publicly available due to privacy of the research participants. The LCBC, DDI and Oslo MCI datasets has restricted access, requests can be made to the corresponding author, and some of the data can be made available given appropriate ethical and data protection approvals.

## Appendix

**Table A1.** Equality of age coefficients on CWIT subtests.

Coefficients to be contrasted	*b* diff	*s.e.*	*Z*	*p*	*b* diff 99% *CI* [LL, UL]
CWIT-1 Age (*b* = −0.049) CWIT-2 Age (*b* = −0.019)	−0.031	0.004	−7.214	<.001	[-0.042, −0.020]
CWIT-1 Age (*b* = −0.049) CWIT-3 Age (*b* = −0.073)	0.024	0.004	5.608	<.001	[0.013, 0.035]
CWIT-1 Age (*b* = −0.049) CWIT-4 Age (*b* = −0.063)	0.014	0.005	2.698	.007	[0.001, 0.027]
CWIT-2 Age (*b* = −0.019) CWIT-3 Age (*b* = −0.073)	0.055	0.005	11.45	<.001	[0.042, 0.067]
CWIT-2 Age (*b* = −0.019) CWIT-4 Age (*b* = −0.063)	0.045	0.005	8.926	<.001	[0.032, 0.057]

*Note. b* = unstandardized beta coefficient; *s.e.* = Standard error of *b* diff.

**Table A2.** Statistics from CWIT subtests on baseline (b) and follow-up (f) for calculating Standard Error of Measurement of the difference for Reliable Change Index (RCI) (*n* = 335).

	*M*	*SD*	Variance	*R*
CWIT-1 (b)	30.26	5.80	33.63	.749
CWIT-1 (f)	30.26	6.11	37.37	
CWIT-2 (b)	21.96	4.26	18.11	.605
CWIT-2 (f)	21.96	4.31	18.54	
CWIT-3 (b)	53.97	13.60	184.87	.841
CWIT-3 (f)	54.04	15.35	235.53	
CWIT-4 (b)	61.99	18.94	358.81	.698
CWIT-4 (f)	62.70	23.02	530.12	

*Note. R* = Pearson’s correlation coefficient between raw scores on CWIT on timepoint 1 and timepoint 2; average test-retest interval between baseline and follow-up was 3.4 years (*SD* = 0.9); CWIT = Color-Word Interference Test.

## References

[CIT0001] Adólfsdóttir, S., Haász, J., Wehling, E., Ystad, M., Lundervold, A., & Lundervold, A. J. (2014). Salient measures of inhibition and switching are associated with frontal lobe gray matter volume in healthy middle-aged and older adults. *Neuropsychology*, *28*(6), 859–869. 10.1037/neu0000082 24819063

[CIT0002] Adólfsdóttir, S., Wollschlaeger, D., Wehling, E., & Lundervold, A. J. (2017). Inhibition and switching in healthy aging: A longitudinal study. *Journal of the International Neuropsychological Society: JINS*, *23*(1), 90–97. 10.1017/S1355617716000898 27938456

[CIT0003] Bayard, S., Erkes, J., & Moroni, C. (2011). Victoria Stroop Test: Normative data in a sample group of older people and the study of their clinical applications in the assessment of inhibition in Alzheimer’s disease. *Archives of Clinical Neuropsychology: The Official Journal of the National Academy of Neuropsychologists*, *26*(7), 653–661. 10.1093/arclin/acr053 21873625

[CIT0005] Ben-David, B. M., Nguyen, L. L., & van Lieshout, P. H. (2011). Stroop effects in persons with traumatic brain injury: Selective attention, speed of processing, or color-naming? A meta-analysis. *Journal of the International Neuropsychological Society: JINS*, *17*(2), 354–363. 10.1017/s135561771000175x 21320377

[CIT0006] Bezdicek, O., Lukavsky, J., Stepankova, H., Nikolai, T., Axelrod, B. N., Michalec, J., Růžička, E., & Kopecek, M. (2015). The Prague Stroop Test: Normative standards in older Czech adults and discriminative validity for mild cognitive impairment in Parkinson’s disease. *Journal of Clinical and Experimental Neuropsychology*, *37*(8), 794–807. 10.1080/13803395.2015.1057106 26313510

[CIT0007] Borghesani, P. R., Madhyastha, T. M., Aylward, E. H., Reiter, M. A., Swarny, B. R., Schaie, K. W., & Willis, S. L. (2013). The association between higher order abilities, processing speed, and age are variably mediated by white matter integrity during typical aging. *Neuropsychologia*, *51*(8), 1435–1444. 10.1016/j.neuropsychologia.2013.03.005 23507612 PMC3875161

[CIT0008] Borland, E., Stomrud, E., van Westen, D., Hansson, O., & Palmqvist, S. (2020). The age-related effect on cognitive performance in cognitively healthy elderly is mainly caused by underlying AD pathology or cerebrovascular lesions: Implications for cutoffs regarding cognitive impairment. *Alzheimer’s Research & Therapy*, *12*(1), 30. 10.1186/s13195-020-00592-8 PMC7093968 32209137

[CIT0009] Bratsberg, B., & Rogeberg, O. (2018). Flynn effect and its reversal are both environmentally caused. *Proceedings of the National Academy of Sciences of the United States of America*, *115*(26), 6674–6678. 10.1073/pnas.1718793115 29891660 PMC6042097

[CIT0010] Brugnolo, A., De Carli, F., Accardo, J., Amore, M., Bosia, L. E., Bruzzaniti, C., Cappa, S. F., Cocito, L., Colazzo, G., Ferrara, M., Ghio, L., Magi, E., Mancardi, G. L., Nobili, F., Pardini, M., Rissotto, R., Serrati, C., & Girtler, N. (2016). An updated Italian normative dataset for the Stroop color word test (SCWT). *Neurological Sciences: Official Journal of the Italian Neurological Society and of the Italian Society of Clinical Neurophysiology*, *37*(3), 365–372. 10.1007/s10072-015-2428-2 26621362

[CIT0011] Clark, L. R., Schiehser, D. M., Weissberger, G. H., Salmon, D. P., Delis, D. C., & Bondi, M. W. (2012). Specific measures of executive function predict cognitive decline in older adults. *Journal of the International Neuropsychological Society: JINS*, *18*(1), 118–127. 10.1017/s1355617711001524 22115028 PMC3314335

[CIT0012] Crawford, J. R., Sutherland, D., & Garthwaite, P. H. (2008). On the reliability and standard errors of measurement of contrast measures from the D-KEFS. *Journal of the International Neuropsychological Society: JINS*, *14*(6), 1069–1073. 10.1017/S1355617708081228 18954487

[CIT0013] de Lange, A.-M G., Bråthen, A. C. S., Rohani, D. A., Fjell, A. M., & Walhovd, K. B. (2018). The temporal dynamics of brain plasticity in aging. *Cerebral Cortex (New York, N.Y.: 1991)*, *28*(5), 1857–1865. 10.1093/cercor/bhy003 29490013 PMC5907343

[CIT0014] Delis, D. (2005). *Delis-Kaplan executive function system, Norwegian version*. Pearson Assessment.

[CIT0015] Delis, D., Kaplan, E., & Kramer, J. (2001). *D-KEFS: Examiners manual*. The Psychological Corporation.

[CIT0016] Duchek, J. M., Balota, D. A., Thomas, J. B., Snyder, A. Z., Rich, P., Benzinger, T. L., Fagan, A. M., Holtzman, D. M., Morris, J. C., & Ances, B. M. (2013). Relationship between stroop performance and resting state functional connectivity in cognitively normal older adults. *Neuropsychology*, *27*(5), 516–528. 10.1037/a0033402 24040929 PMC3837537

[CIT0017] Egeland, J., Løvstad, M., Norup, A., Nybo, T., Persson, B. A., Rivera, D. F., Schanke, A.-K., Sigurdardottir, S., & Arango-Lasprilla, J. C. (2016). Following international trends while subject to past traditions: Neuropsychological test use in the Nordic countries. *The Clinical Neuropsychologist*, *30*(sup1), 1479–1500. 10.1080/13854046.2016.1237675 27670676

[CIT0018] Espenes, J., Hessen, E., Eliassen, I. V., Waterloo, K., Eckerström, M., Sando, S. B., Timón, S., Wallin, A., Fladby, T., & Kirsebom, B.-E. (2020). Demographically adjusted trail making test norms in a Scandinavian sample from 41 to 84 years. *The Clinical Neuropsychologist*, *34*(sup1), 110–126. 10.1080/13854046.2020.1829068 33034252

[CIT0019] Fagerland, M. W., Lydersen, S., & Laake, P. (2014). Recommended tests and confidence intervals for paired binomial proportions. *Statistics in Medicine*, *33*(16), 2850–2875. 10.1002/sim.6148 24648355

[CIT0020] Ferro, A. M., Brugnolo, A., De Leo, C., Dessi, B., Girtler, N., Morbelli, S., Nobili, F., Rossi, D. S., Falchero, M., Murialdo, G., Rossini, P. M., Babiloni, C., Schizzi, R., Padolecchia, R., & Rodriguez, G. (2005). Stroop interference task and single-photon emission tomography in anorexia: A preliminary report. *The International Journal of Eating Disorders*, *38*(4), 323–329. 10.1002/eat.20203 16231338

[CIT0021] Fjell, A. M., Chen, C.-H., Sederevicius, D., Sneve, M. H., Grydeland, H., Krogsrud, S. K., Amlien, I., Ferschmann, L., Ness, H., Folvik, L., Beck, D., Mowinckel, A. M., Tamnes, C. K., Westerhausen, R., Håberg, A. K., Dale, A. M., & Walhovd, K. B. (2019). Continuity and discontinuity in human cortical development and change from embryonic stages to old age. *Cerebral Cortex (New York, N.Y.: 1991)*, *29*(9), 3879–3890. 10.1093/cercor/bhy266 30357317

[CIT0022] Fjell, A. M., Sneve, M. H., Grydeland, H., Storsve, A. B., & Walhovd, K. B. (2017). The disconnected brain and executive function decline in aging. *Cerebral Cortex (New York, N.Y.: 1991)*, *27*(3), 2303–2317. 10.1093/cercor/bhw082 27073220

[CIT0023] Fladby, T., Pålhaugen, L., Selnes, P., Waterloo, K., Bråthen, G., Hessen, E., Almdahl, I. S., Arntzen, K.-A., Auning, E., Eliassen, C. F., Espenes, R., Grambaite, R., Grøntvedt, G. R., Johansen, K. K., Johnsen, S. H., Kalheim, L. F., Kirsebom, B.-E., Müller, K. I., Nakling, A. E., … Aarsland, D. (2017). Detecting at-risk Alzheimer’s disease cases. *Journal of Alzheimer’s Disease: JAD*, *60*(1), 97–105. 10.3233/JAD-170231 28826181 PMC5611830

[CIT0025] Gelman, A., & Stern, H. (2006). The difference between "significant" and "not significant" is not itself statistically significant. *The American Statistician*, *60*(4), 328–331. 10.1198/000313006X152649

[CIT0026] Guhn, M., Forer, B., Zumbo, B. D. (2014). Reliable Change Index. In: Michalos, A.C. (Eds) Encyclopedia of Quality of Life and Well-Being Research. Springer, Dordrecht. 10.1007/978-94-007-0753-5_2465

[CIT0027] Guilmette, T. J., Sweet, J. J., Hebben, N., Koltai, D., Mahone, E. M., Spiegler, B. J., Stucky, K., & Westerveld, M. (2020). American Academy of Clinical Neuropsychology consensus conference statement on uniform labeling of performance test scores. *The Clinical Neuropsychologist*, *34*(3), 437–453. 10.1080/13854046.2020.1722244 32037942

[CIT0028] Hankee, L. D., Preis, S. R., Piers, R. J., Beiser, A. S., Devine, S. A., Liu, Y., Seshadri, S., Wolf, P. A., & Au, R. (2016). Population normative data for the CERAD word list and victoria stroop test in younger- and middle-aged adults: Cross-sectional analyses from the framingham heart study. *Experimental Aging Research*, *42*(4), 315–328. 10.1080/0361073X.2016.1191838 27410241 PMC4946576

[CIT0029] Harrington, K. D., Schembri, A., Lim, Y. Y., Dang, C., Ames, D., Hassenstab, J., Laws, S. M., Rainey-Smith, S., Robertson, J., Rowe, C. C., Sohrabi, H. R., Salvado, O., Weinborn, M., Villemagne, V. L., Masters, C. L., & Maruff, P. (2018). Estimates of age-related memory decline are inflated by unrecognized Alzheimer’s disease. *Neurobiology of Aging*, *70*, 170–179. 10.1016/j.neurobiolaging.2018.06.005 30015036

[CIT0030] Hasher, L., & Zacks, R. T. (1988). Working memory, comprehension, and aging: A review and a new view. *Psychology of Learning and Motivation*, *22*, 193–225.

[CIT0031] Heaton, R. K., Avitable, N., Grant, I., & Matthews, C. G. (1999). Further crossvalidation of regression-based neuropsychological norms with an update for the Boston Naming Test. *Journal of Clinical and Experimental Neuropsychology*, *21*(4), 572–582. 10.1076/jcen.21.4.572.882 10550815

[CIT0032] Henningsen, A., & Hamann, J. D. (2007). systemfit: A package for estimating systems of simultaneous equations in R. *Journal of Statistical Software*, *23*(4), 1–40. 10.18637/jss.v023.i04

[CIT0033] Hessel, P., Kinge, J. M., Skirbekk, V., & Staudinger, U. M. (2018). Trends and determinants of the Flynn effect in cognitive functioning among older individuals in 10 European countries. *Journal of Epidemiology and Community Health*, *72*(5), 383–389. https://jech.bmj.com/content/jech/72/5/383.full.pdf. 10.1136/jech-2017-209979 29440306

[CIT0034] Hothorn, T., Bretz, F., Westfall, P., Heiberger, R. M., Schuetzenmeister, A., Scheibe, S., & Hothorn, M. T. (2016). Package ‘multcomp’. In *Simultaneous Inference in General Parametric Models. Project for Statistical Computing*, Vienna, Austria.

[CIT0035] International Test Commision. (2001). International guidelines for test use. *International Journal of Testing*, *1*(2), 93–114. 10.1207/S15327574IJT0102_1 10.1207/S15327574IJT0102_1

[CIT0036] Jager, J., Putnick, D. L., & Bornstein, M. H. (2017). II. More than just convenient: The scientific merits of homogeneous convenience samples. *Monographs of the Society for Research in Child Development*, *82*(2), 13–30. 10.1111/mono.12296 28475254 PMC5606225

[CIT0037] James, G., Witten, D., Hastie, T., & Tibshirani, R. (2021). *An introduction to statistical learning: with applications in R*. Springer.

[CIT0038] Keifer, E., & Tranel, D. (2013). A neuropsychological investigation of the Delis-Kaplan executive function system. *Journal of Clinical and Experimental Neuropsychology*, *35*(10), 1048–1059. 10.1080/13803395.2013.854319 24236952 PMC4304768

[CIT0039] Kirsebom, B.-E., Espenes, R., Hessen, E., Waterloo, K., Johnsen, S. H., Gundersen, E., Botne Sando, S., Rolfseng Grøntvedt, G., Timón, S., & Fladby, T. (2019). Demographically adjusted CERAD wordlist test norms in a Norwegian sample from 40 to 80 years. *The Clinical Neuropsychologist*, *33*(sup1), 27–39. 10.1080/13854046.2019.1574902 30849268

[CIT0040] Koo, T. K., & Li, M. Y. (2016). A guideline of selecting and reporting intraclass correlation coefficients for reliability research. *Journal of Chiropractic Medicine*, *15*(2), 155–163. 10.1016/j.jcm.2016.02.012 27330520 PMC4913118

[CIT0042] Ktaiche, M., Fares, Y., & Abou-Abbas, L. (2022). Stroop color and word test (SCWT): Normative data for the Lebanese adult population. *Applied Neuropsychology. Adult*, *29*(6), 1578–1586. 10.1080/23279095.2021.1901101 33780300

[CIT0043] Lippa, S. M., & Davis, R. N. (2010). Inhibition/switching is not necessarily harder than inhibition: An analysis of the D-KEFS Color-Word Interference Test. *Archives of Clinical Neuropsychology: The Official Journal of the National Academy of Neuropsychologists*, *25*(2), 146–152. 10.1093/arclin/acq001 20139109

[CIT0044] Magnus, P., Irgens, L. M., Haug, K., Nystad, W., Skjaerven, R., & Stoltenberg, C. (2006). Cohort profile: The Norwegian mother and child cohort study (MoBa). *International Journal of Epidemiology*, *35*(5), 1146–1150. 10.1093/ije/dyl170 16926217

[CIT0045] Magnusdottir, B., Haraldsson, H., & Sigurdsson, E. (2021). Trail making test, stroop, and verbal fluency: Regression-based norms for the icelandic population. *Archives of Clinical Neuropsychology: The Official Journal of the National Academy of Neuropsychologists*, *36*(2), 253–266. 10.1093/arclin/acz049 31732743

[CIT0046] Milham, M. P., Erickson, K. I., Banich, M. T., Kramer, A. F., Webb, A., Wszalek, T., & Cohen, N. J. (2002). Attentional control in the aging brain: Insights from an fMRI study of the stroop task. *Brain and Cognition*, *49*(3), 277–296. https://www.sciencedirect.com/science/article/pii/S0278262601915015. 10.1006/brcg.2001.1501 12139955

[CIT0047] Miller, E. K., & Cohen, J. D. (2001). An integrative theory of prefrontal cortex function. *Annual Review of Neuroscience*, *24*(1), 167–202. https://www.annualreviews.org/doi/abs/10.1146/annurev.neuro.24.1.167. 10.1146/annurev.neuro.24.1.167 11283309

[CIT0048] Mitrushina, M., Boone, K. B., Razani, J., & D’Elia, L. F. (2005). *Handbook of normative data for neuropsychological assessment*. : Oxford University Press.

[CIT0049] Peña-Casanova, J., Quiñones-Ubeda, S., Gramunt-Fombuena, N., Quintana, M., Aguilar, M., Molinuevo, J. L., Serradell, M., Robles, A., Barquero, M. S., Payno, M., Antúnez, C., Martínez-Parra, C., Frank-García, A., Fernández, M., Alfonso, V., Sol, J. M., & Blesa, R. (2009). Spanish Multicenter Normative Studies (NEURONORMA Project): Norms for the Stroop color-word interference test and the Tower of London-Drexel. *Archives of Clinical Neuropsychology: The Official Journal of the National Academy of Neuropsychologists*, *24*(4), 413–429. 10.1093/arclin/acp043 19661108

[CIT0050] Rabin, L. A., Barr, W. B., & Burton, L. A. (2005). Assessment practices of clinical neuropsychologists in the United States and Canada: A survey of INS, NAN, and APA Division 40 members. *Archives of Clinical Neuropsychology: The Official Journal of the National Academy of Neuropsychologists*, *20*(1), 33–65. 10.1016/j.acn.2004.02.005 15620813

[CIT0051] Regard, M. (1981). *Stroop test–Victoria version*. Neuropsychological Laboratory, University of Victoria.

[CIT0052] Revelle, W. (2023). *psych: Procedures for Psychological, Psychometric, and Personality Research*. Northwestern University, Evanston, Illinois. R package version 2.3.9, https://CRAN.R-project.org/package=psych.

[CIT0053] Ritchie, S. J., & Tucker-Drob, E. M. (2018). How much does education improve intelligence? A meta-analysis. *Psychological Science*, *29*(8), 1358–1369. 10.1177/0956797618774253 29911926 PMC6088505

[CIT0055] Ryder, T. (2021). Testkvalitetsprosjektet-del 1: Norske psykologers testholdninger og testbruk. *Tidsskrift for Norsk Psykologforening*, *58*(1), 28–37.

[CIT0056] Salthouse, T. A. (1996). The processing-speed theory of adult age differences in cognition. *Psychological Review*, *103*(3), 403–428. 10.1037/0033-295X.103.3.403 8759042

[CIT0057] Scarpina, F., & Tagini, S. (2017). The stroop color and word test. *Frontiers in Psychology*, *8*, 557. 10.3389/fpsyg.2017.00557 28446889 PMC5388755

[CIT0058] Seo, E. H., Lee, D. Y., Choo, I. H., Kim, S. G., Kim, K. W., Youn, J. C., Jhoo, J. H., & Woo, J. I. (2008). Normative study of the Stroop Color and Word Test in an educationally diverse elderly population. *International Journal of Geriatric Psychiatry*, *23*(10), 1020–1027. 10.1002/gps.2027 18425990

[CIT0059] Shrout, P. E., & Fleiss, J. L. (1979). Intraclass correlations: Uses in assessing rater reliability. *Psychological Bulletin*, *86*(2), 420–428. 10.1037//0033-2909.86.2.420 18839484

[CIT0060] Sjoberg, E. A., Wilner, R. G., D’Souza, A., & Cole, G. G. (2023). The Stroop task sex difference: Evolved inhibition or color naming? *Archives of Sexual Behavior*, *52*(1), 315–323. 10.1007/s10508-022-02439-9 36261735 PMC9859918

[CIT0061] Skirbekk, V., Stonawski, M., Bonsang, E., & Staudinger, U. M. (2013). The Flynn effect and population aging. *Intelligence*, *),* *41*(3), 169–177. 10.1016/j.intell.2013.02.001

[CIT0062] Statistics Norway. (2022). *Facts about education in Norway 2022 - key figures 2020*. https://www.ssb.no/en/utdanning/utdanningsniva/artikler/facts-about-education-in-norway-2022/_/attachment/inline/5ea7e453-8d7c-4423-80fb-b8bd2b8a0340:10c4b259c03df8c44ee58708542c2cf7977a9b4a/FOU-2022-web-en.pdf

[CIT0063] Steinberg, B. A., Bieliauskas, L. A., Smith, G. E., & Ivnik, R. J. (2005). Mayo’s older Americans normative studies: Age- and IQ-adjusted norms for the Trailmaking Test, the Stroop Test, and Mae Controlled Oral Word Association Test. *The Clinical Neuropsychologist*, *19*(3–4), 329–377. 10.1080/13854040590945210 16120535

[CIT0066] Stern, Y., Albert, M., Barnes, C. A., Cabeza, R., Pascual-Leone, A., & Rapp, P. R. (2023). A framework for concepts of reserve and resilience in aging. *Neurobiology of Aging*, *124*, 100–103. 10.1016/j.neurobiolaging.2022.10.015 36653245 PMC10424718

[CIT0067] Storsve, A. B., Fjell, A. M., Tamnes, C. K., Westlye, L. T., Overbye, K., Aasland, H. W., & Walhovd, K. B. (2014). Differential longitudinal changes in cortical thickness, surface area and volume across the adult life span: Regions of accelerating and decelerating change. *The Journal of Neuroscience: The Official Journal of the Society for Neuroscience*, *34*(25), 8488–8498. 10.1523/JNEUROSCI.0391-14.2014 24948804 PMC6608217

[CIT0068] Streeter, C. C., Terhune, D. B., Whitfield, T. H., Gruber, S., Sarid-Segal, O., Silveri, M. M., Tzilos, G., Afshar, M., Rouse, E. D., Tian, H., Renshaw, P. F., Ciraulo, D. A., & Yurgelun-Todd, D. A. (2008). Performance on the Stroop predicts treatment compliance in cocaine-dependent individuals. *Neuropsychopharmacology: Official Publication of the American College of Neuropsychopharmacology*, *33*(4), 827–836. 10.1038/sj.npp.1301465 17568399

[CIT0069] Stuss, D. T., Floden, D., Alexander, M. P., Levine, B., & Katz, D. (2001). Stroop performance in focal lesion patients: Dissociation of processes and frontal lobe lesion location. *Neuropsychologia*, *39*(8), 771–786. 10.1016/s0028-3932(01)00013-6 11369401

[CIT0070] Tamnes, C. K., Walhovd, K. B., Dale, A. M., Østby, Y., Grydeland, H., Richardson, G., Westlye, L. T., Roddey, J. C., Hagler, D. J., Due-Tønnessen, P., Holland, D., & Fjell, A. M. (2013). Brain development and aging: Overlapping and unique patterns of change. *Neuroimage*, *68*, 63–74. 10.1016/j.neuroimage.2012.11.039 23246860 PMC5378867

[CIT0071] Testa, S. M., Winicki, J. M., Pearlson, G. D., Gordon, B., & Schretlen, D. J. (2009). Accounting for estimated IQ in neuropsychological test performance with regression-based techniques. *Journal of the International Neuropsychological Society: JINS*, *15*(6), 1012–1022. 10.1017/s1355617709990713 19796440

[CIT0072] Trahan, L. H., Stuebing, K. K., Fletcher, J. M., & Hiscock, M. (2014). The Flynn effect: A meta-analysis. *Psychological Bulletin*, *140*(5), 1332–1360. 10.1037/a0037173 24979188 PMC4152423

[CIT0073] Tremblay, M.-P., Potvin, O., Belleville, S., Bier, N., Gagnon, L., Blanchet, S., Domingues, N.-S., Gaudreau, G., Macoir, J., & Hudon, C. (2016). The victoria stroop test: Normative data in Quebec-French adults and elderly. *Archives of Clinical Neuropsychology: The Official Journal of the National Academy of Neuropsychologists*, *31*(8), 926–933. 10.1093/arclin/acw029 27246959 PMC5859918

[CIT0074] Troyer, A. K., Leach, L., & Strauss, E. (2006). Aging and response inhibition: Normative data for the Victoria Stroop Test. *Neuropsychology, Development, and Cognition. Section B, Aging, Neuropsychology and Cognition*, *13*(1), 20–35. 10.1080/138255890968187 16766341

[CIT0075] Van der Elst, W., Van Boxtel, M. P., Van Breukelen, G. J., & Jolles, J. (2006). The Stroop color-word test: Influence of age, sex, and education; and normative data for a large sample across the adult age range. *Assessment*, *13*(1), 62–79. 10.1177/1073191105283427 16443719

[CIT0076] Wickham, H., François, R., Henry, L., & Müller, K. (2022). *dplyr: A grammar ofdata manipulation*. R package version1. 0.8. https://CRAN. R-project. org/package=dplyr.

[CIT0077] Willse, J. T. (2022). *CTT: Classical Test Theory Functions*. R package version 2.3.3. https://rdrr.io/cran/CTT/

[CIT0078] Wood, S., & Wood, M. S. (2015). Package ‘mgcv’. *R Package Version*, *1*(29), 729.

[CIT0080] Yu, L., Boyle, P. A., Segawa, E., Leurgans, S., Schneider, J. A., Wilson, R. S., & Bennett, D. A. (2015). Residual decline in cognition after adjustment for common neuropathologic conditions. *Neuropsychology*, *29*(3), 335–343. 10.1037/neu0000159 25495832 PMC4420708

[CIT0081] Zalonis, I., Christidi, F., Bonakis, A., Kararizou, E., Triantafyllou, N. I., Paraskevas, G., Kapaki, E., & Vasilopoulos, D. (2009). The stroop effect in Greek healthy population: Normative data for the Stroop Neuropsychological Screening Test. *Archives of Clinical Neuropsychology: The Official Journal of the National Academy of Neuropsychologists*, *24*(1), 81–88. 10.1093/arclin/acp011 19395358

